# Annual acknowledgement of manuscript reviewers

**DOI:** 10.1186/1471-2199-14-8

**Published:** 2013-03-28

**Authors:** Christopher Morrey

**Affiliations:** 1 BioMed Central, Floor 6, 236 Gray's Inn Road, London, WC1X 8HB, UK

## Abstract

**Contributing reviewers:**

The editors of BMC Molecular Biology would like to thank all of our reviewers who have contributed to the journal in Volume 12 (2012).

## 

Abalo Chango

France

Alan Grossman

USA

Alessandro Granito

Italy

Alex Bishop

USA

Alexander Dobrovic

Australia

Anbin Hu

China

Arie Vaandrager

Netherlands

Ashley Monks

Canada

Baruch Frenkel

USA

Bernd Knoll

Germany

Blanca Rubi

Spain

Bo Thomsen

Denmark

Brad Coates

USA

Burckhard Seelig

USA

Camiel Wielders

Netherlands

Carla Oliveira

Brazil

Catherine Putonti

USA

Charles Query

USA

Claudio Hetz

Chile

Cyril Sanders

UK

David Dryden

UK

David McPheeters

USA

David Peter Hornby

UK

Dawn Chandler

USA

Dezso Peter Virok

Hungary

Dimitri Pestov

USA

Dirk Motzkus

Germany

Dominique Gagliardi

France

Edward Bolt

UK

Elaine Sia

USA

Emanuele Buratti

Italy

Eric Peatman

USA

Eric Deprez

France

Eulalia De Nadal

Spain

Evdokia Pasheva

Bulgaria

Evgeny Zdobnov

Switzerland

Fei Li

China

Fernando Corrales

Spain

François Lefèvre

France

Francoise Bernerd

France

Frank Van Steenbeek

Netherlands

Gary Hausman

USA

Gary Halliday

Australia

Gerald Wyckoff

USA

Gerald Thiel

Germany

Giacomo Spinsanti

Italy

Gilbert Cote

USA

Greg Shipley

USA

Han-Fu Cheng

Taiwan

Hans Lehrach

Germany

Hasan Khatib

USA

Hee Jeong Kong

South Korea

Helder Nakaya

USA

Hendrik Viljoen

USA

Huiling Cao

USA

Idriss Bennani-Baiti

Austria

Igor Vorechovsky

UK

Ismail Zaitoun

USA

Jacob Seeler

France

Jaewhan Song

South Korea

James Mulloy

USA

James Brooks

USA

Jan Roelof Van Der Meer

Switzerland

Jane Flint

USA

Jasmina Dikic

Germany

Jean Dufer

France

Jiexiong Feng

China

Jim Huggett

UK

Jirong Long

USA

Johan Ledin

Sweden

Joost Martens

Netherlands

Jörg R. Aschenbach

Germany

Joseph Dillard

USA

Kai Kristoffer Lie

Norway

Kajal Biswas

USA

Kelly Mcnagny

Canada

Kelvin Li

USA

Kuniaki Saito

Japan

Luis Afonso

Australia

Lukasz Jaskiewicz

Switzerland

Manju Hingorani

USA

Manuel Ares

USA

Maria Jasin

USA

Marisa Medina

USA

Martin Marinus

USA

Matthias Duerst

Germany

Maurizio Callari

Italy

Michael Mceachern

USA

Michael O'Connor

USA

Min-Hao Kuo

USA

Minsheng You

China

Mitch Mcvey

USA

Momtchil Vodenitcharov

Canada

Mounira Amor-Guéret

France

Mu-Shui Dai

USA

Nicholas Redshaw

UK

Nina Le Bert

Singapore

Nobuyuki Takei

Japan

Penka Nikolova

UK

Peter Vallone

USA

Petra Imhof

Germany

Petras Dzeja

USA

Philip Sharp

USA

Photini-Effie Tsilibary

Greece

Piero Bianco

USA

Prakash Kulkarni

USA

Qingjun Wu

China

Ramesh Pillai

France

Ran Blekhman

USA

Roger Reddel

Australia

Samir Acharya

USA

Samir El Andaloussi

UK

Satoshi Kida

Japan

Shankar Pattabhiraman

Germany

Sheng-Chung Lee

Taiwan

Silvia Moncini

Italy

Simon Alberti

Germany

Sirawut Klinbunga

Thailand

Sissades Tongsima

Thailand

Smita Mohanty

USA

Sonja Maria Walzer

Austria

Stefan Stamm

Germany

Steinunn Baekkeskov

USA

Steve Stowers

USA

Steven Matson

USA

Suming Huang

USA

Suresh Cuddapah

USA

Susan Lindquist

USA

Susan Chan

France

Taisen Iguchi

Japan

Takafumi Noma

Japan

Tien-Jye Chang

Taiwan

Urs Albrecht

Switzerland

Valer Gotea

USA

Vito Cicinnati

Germany

Wensheng Yan

USA

Werner Bergen

USA

William Nelson

USA

Xin Chen

Taiwan

Xuguo Zhou

USA

Yihong Ye

USA

Yoav Arava

Israel

Yong Sam Jung

USA

Yuchio Yanagawa

Japan

Zhen Zhang

China

